# Efficacy and Safety of Tofacitinib in Rheumatoid Arthritis (RA): A Retrospective Study From Two Centers in Jeddah, Saudi Arabia

**DOI:** 10.7759/cureus.32240

**Published:** 2022-12-05

**Authors:** Zeyad Alzahrani, Ahmed Alhazmi, Hanan Almalki, Noura Aljehani, Mohammed Dumyati, Hadeel Alabdali

**Affiliations:** 1 Rheumatology, National Guard Health Affairs, King Abdulaziz Medical City, Jeddah, SAU; 2 Rheumatology, King Fahad Armed Forces Hospital, Jeddah, SAU; 3 Internal Medicine, National Guard Health Affairs, King Abdulaziz Medical City, Jeddah, SAU; 4 Internal Medicine, King Abdulaziz Hospital, Mecca, SAU

**Keywords:** rheumatoid arthritis, tofacitinib, remission, low disease activity, safety

## Abstract

Background: Tofacitinib is the first Janus kinase (JAK) inhibitor approved for treating rheumatoid arthritis (RA). Several clinical trials have evaluated the safety and effectiveness of tofacitinib in adult patients with moderately to severely active RA. Real-world studies provide invaluable insights into routine clinical practice. We aim to assess the clinical efficacy and safety of RA patients.

Methods: Over a period of two years, we included 50 consecutive RA patients who were treated with tofacitinib. Clinical disease activity, assessed by disease activity score (DAS) 28 - erythrocyte sedimentation rate (ESR), as well as adverse events (AEs) were evaluated.

Results: A total of 50 patients (84% female) were enrolled in the study. The mean age at initiation of tofacitinib was 48.54 ± 15.97 years. The mean time of treatment with tofacitinib was 18.06 ± 2.04 months. Patients were treated with tofacitinib 5 mg BID with 32% receiving tofacitinib as monotherapy. A total of 74% of the patients had been prescribed at least one biological treatment. The treatment target was achieved in 42 patients (82%). Baseline characteristics and previous treatment regimens did not predict clinical response to tofacitinib. Fifteen patients discontinued the treatment: seven due to ineffectiveness, four due to pregnancy, and five due to adverse events. The most common infectious adverse event was herpes zoster (4%) while the most common observed laboratory abnormalities were elevation in low density lipoprotein (LDL) and high density lipoprotein (HDL) in 6% and 8% of the patients, respectively.

Conclusion: Our results indicate that tofacitinib is effective in real-world settings even as monotherapy. The treatment target was attained by 82% of the patients on tofacitinib. The safety profile of tofacitinib was generally consistent with previous studies.

## Introduction

Rheumatoid arthritis (RA) is a systemic autoimmune disease characterized by chronic inflammation and the destruction of joints. It can lead to functional impairment, disability, and reduced quality of life [1].

The 2019 European League Against Rheumatism (EULAR) treatment guidelines recommend “treat-to-target” strategy (T2T) with early and intensive treatment using disease-modifying anti-rheumatic drugs (DMARDs) [2]. T2T strategy’s purpose is to achieve remission or low disease activity. Methotrexate (MTX), a conventional synthetic DMARD (csDMARD), has long been the cornerstone of treatment [2]. A biological DMARD (bDMARD) or a Janus kinase (JAK) inhibitor should be implemented after the failure of initial csDMARDs in the presence of poor prognostic factors [2].

Tofacitinib is a JAK inhibitor intended for the treatment of moderately to severely active RA in adults who failed or are intolerant to csDMARDs [3]. Tofacitinib, an orally administered small molecule, may provide a convenient benefit over injectable bDMARDs [4]. Clinical trials have demonstrated that tofacitinib, either as monotherapy or in conjunction with csDMARDs, is able to considerably reduce disease activity as measured by the American College of Rheumatology (ACR) response rates, the EULAR responses and Health Assessment Questionnaire-Disability Index (HAQ-DI) scores [5]. Studies comparing tofacitinib with other therapeutic approaches in the treatment of RA propose that the effectiveness of tofacitinib is comparable to that of biological agents [6].

In 2012, tofacitinib was approved by the Food and Drug Administration (FDA) for adult patients with moderate to severe RA who have previously failed to respond to MTX [7]. Additionally, the European Medicines Agency (EMA) granted approval in 2017 [8].

The U.S. Corrona RA registry reported that tofacitinib and bDMARD initiators had a similar incidence rate of adverse events including major adverse cardiovascular events (MACE), serious infection, malignancy, death, and venous thromboembolism (VTE) between tofacitinib and bDMARDs. However, herpes zoster was significantly higher with tofacitinib [9].

Recently, ORAL Surveillance, which was mandated by the U.S. FDA reported higher incidence rates of MACE and malignancy than tumor necrosis factor (TNF) inhibitors. The risk of opportunistic infections (including herpes zoster and tuberculosis), abnormalities on the liver-function tests, and serum lipid levels were more frequent with tofacitinib than with a TNF inhibitor [10].

Because JAK inhibitors are still a relatively new therapeutic option for RA, further clinical experience with tofacitinib is required to further evaluate its safety and efficacy. Therefore, it is of clinical importance to further investigate the effects of tofacitinib, particularly when administered as monotherapy to RA patients. 

This study aims to assess the clinical efficacy, safety, and tolerable response to tofacitinib in RA patients encountered in routine clinical practice. To our knowledge, this is the first study evaluating the efficacy of tofacitinib in RA patients in Saudi Arabia.

## Materials and methods

Subjects and methods

This is a record-based retrospective study conducted at the National Guard Hospital (NGH) and King Fahad Armed Forces Hospital (KFAFH), Jeddah, Saudi Arabia over a period of two years from January 2020 to January 2022. The participants were diagnosed with RA according to ACR/EULAR 2010 classification criteria [11] and were seen in the outpatient clinics at NGH and KFAFH.

The inclusion criteria were all RA patients aged ≥18 years, who were treated with tofacitinib between 2015 and 2022 and were evaluated at the initial visit (baseline) and every six months thereafter. The exclusion criteria were any patient aged younger than 18 years and those with a recent start on tofacitinib (less than one month).

Patients' records were reviewed for the following information at the start of treatment with tofacitinib: age, gender, comorbid illnesses, disease duration, rheumatoid factor (RF) and anti-citrulline protein antibody (anti-CCP) positivity, presence of erosions and extra-articular manifestation, history of previous DMARDs, and concomitant medications. Clinical disease activity was assessed using disease activity score (DAS) 28 -- ESR for RA [12]. Disease activity was categorized as follows: remission (<2.6), low disease activity (LDA) (2.6-3.2), moderate disease activity (MDA) (>3.2-5.1), and high disease activity (HDA) (>5.1).

Data on medication safety including reasons of discontinuation (if any) and adverse events in the form of MACE, malignancy, serious infection requiring hospitalization, VTE, and herpes zoster were documented. The following laboratory tests were reviewed: hemoglobin (g/dL), platelets (mm3) leukocytes (mm3), lipid profile, serum creatinine, alanine aminotransferase (IU/L), and aspartate aminotransferase (IU/L).

Our study adheres to Helsinki Declaration principles and is approved by the research ethics committee of the NGH and KFAFH. Informed patient consents were not required as there was no direct influence on the participants.

Data analysis

We analyzed data using SPSS version 26 (IBM Corp., Armonk, NY). The qualitative data were expressed as numbers and percentages, whereas the quantitative data were expressed as mean and standard deviation (Mean SD). The Chi-squared test (2) was used to test the relationship between variables. The Mann-Whitney and Kruskal Wallis tests were used to examine non-parametric variables. A statistically significant p-value was less than 0.05.

## Results

We studied a total of 50 patients. Baseline demographic and clinical characteristics are represented in Table 1. The mean age at initiation of tofacitinib was 48.54 ± 15.97 years, and 84% were females. The mean duration of the disease was 10.68 ± 6.87 years. A significant proportion of patients had comorbidities (66%). Obesity (body mass index, BMI > 30), hypertension, and diabetes mellitus were prevalent in 47.0%, 30.1%, and 13.0% of patients, respectively. Eighty-six percent of the patients were positive for RF or anti-CCP and 30% had evidence of radiographic erosions. Extra-articular manifestations were found in seven (14%) of the patients, including rheumatoid nodules in two patients, secondary Sjogren syndrome in two patients, interstitial lung disease in two patients, and episcleritis in one. Patients included in the study had moderate (68%) and high (32%) disease activity at baseline according to DAS 28 -- ESR.

**Table 1:** Baseline patient sociodemographic and clinical characteristics. SD, standard deviation; BMI, body mass index; DM, diabetes mellites; HTN, hypertension; IHD, ischemic heart disease; RF, rheumatoid factor; anti-CCP, anti-citrulline protein antibody

Variable	All patients
	(n = 50)
Age at initiation of tofacitinib, years (mean ± SD)	48.54 ± 15.97
Duration of disease, years (mean ± SD)	10.68 ± 6.87
Gender	
Female	42 (84)
Male	8 (16)
BMI	
< 24.9	10 (20%)
25-29.9	16 (32%)
≥ 30	24 (48%)
Comorbidities	
None	17 (34%)
DM	16 (32%)
HTN	24 (48%)
IHD	2 (4%)
Dyslipidemia	13 (26%)
Others	14 (28%)
Positive RF or CCP	
No	7 (14)
Yes	43 (86)
Presence of erosion	
No	35 (70)
Yes	15 (30)
Extra-articular manifestations	
No	43 (86)
Yes	7 (14)
Disease activity at baseline	
Moderate disease activity	34 (68)
Severe disease activity	16 (32)

All patients were initiated on tofacitinib at 5 mg twice daily of which 32% received tofacitinib as monotherapy. Conventional synthetic DMARDs were taken concurrently with tofacitinib by 68% of the patients while 42% were on oral steroids at doses ranging from 2.5 to 7.5 mg. The current treatment regimen is detailed in Table 2.

**Table 2:** Treatment regimen. csDMARDs, conventional synthetic disease modifying anti-rheumatic drugs; bDMARDs, biological disease modifying anti-rheumatic drugs

Variable	All patients
	(n = 50)
Tofacitinib as monotherapy	
No	34 (68)
Yes	16 (32)
Concomitant csDMARDs	
Hydroxychloroquine	4 (8)
Methotrexate	28 (56)
Sulfasalazine	2 (4)
None	18 (32)
Concomitant prednisolone	21 (42)
Given after a biological agent	
No	13 (26)
Yes	37 (74)
Number of previous csDMARDs	1.5 ± 0.58
Number of previous bDMARDs	1.34 ± 1.9

A total of 74% of the patients had been previously exposed to at least one biological agent. The overall number of patients who had been prescribed one, two, three, or more biologics were 16, 14, and 7, respectively. During the evaluation, 70% of patients were still on tofacitinib, the mean duration of therapy was 18.06 ± 2.04 months. In our study, remission was observed in half of the patients and low-disease activity was achieved in 30%. The time to achieve the treatment target was between 12 and 26 weeks.

During follow-up, tofacitinib was discontinued in 15 (30%) patients. A total of seven patients stopped tofacitinib due to insufficient response, four due to pregnancy, and five due to adverse events. Herpes zoster was developed in two patients after starting tofacitinib, two patients developed serious infections requiring hospitalization (complicated urinary tract infection, UTI and pneumonia), and a patient developed neutropenia less than 1000/mm3. It should be noted that the herpes zoster vaccine was not available at that time in Saudi Arabia. For patients who discontinued treatment because of infection, tofacitinib was resumed after infection resolution while patients with herpes zoster discontinued tofacitinib permanently. Herpes zoster infections occurred 8-16 weeks after starting tofacitinib. None of the studied patients developed serious adverse events (VTE, MACE, or malignancy) during the follow-up period. The reasons for treatment discontinuation are summarized and shown in Table 3.

**Table 3:** Treatment discontinuation. SD, standard deviation

Variable	All patients
	(n = 50)
Total duration of therapy, months (mean ± SD)	18.06 ± 2.04
Discontinuation of tofacitinib	
No	35 (70)
Yes	15 (30)
Reason of discontinuation?	
Lack of efficacy	7 (14)
Pregnancy	4 (8)
Adverse events	5 (10)
Adverse events	
Dyslipidemia	3 (6)
Herpes zoster	2 (4)
Serious infection requiring hospitalization	2 (4)
Neutropenia	1 (2)

Laboratory values including hemoglobin, white blood cell counts, liver enzymes, creatinine, and lipid profile were followed during tofacitinib treatment, as shown in Table 4. Among the studied subjects, 12% had decreased hemoglobin levels of less than 2 g, while only 2% had neutropenia. An increase in cholesterol or low density lipoprotein (LDL) level was detected in 6% and increased high density lipoprotein (HDL) in 8%. None of the studied patients developed lymphopenia, an increase in liver enzymes, or creatinine.

**Table 4:** Laboratory changes. Hb, hemoglobin; TG, triglyceride; LDL, low density lipoprotein; HDL, high density lipoprotein

Variable	All patients
	(n = 50)
Anemia (decreased Hb by > 1 g)	6 (12)
Leukopenia (<4000 / mm3)	1 (2)
Neutropenia (<1500 / mm3)	1 (2)
Lipid profile	
Elevation in TG	1 (2)
Elevation in total cholesterol	3 (6)
Elevation in LDL	3 (6)
Elevation in HDL	4 (8)

The predictors of clinical response to tofacitinib are shown in Table 5. No significant relationship was found between tofacitinib efficacy and patients' demographics at baseline, disease characteristics, or previous treatment regimen.

**Table 5:** Predictors of achievement of the treatment target. BMI, body mass index; csDMARDs, conventional synthetic disease-modifying anti-rheumatic drugs; bDMARDs, biological disease-modifying anti-rheumatic drugs; χ2, Chi-square

	Efficacy		χ2	p-value
Variable	Did not achieve the therapy goal	Achieved the therapy goal No. (%)		
	No. (%)			
Age at initiation of tofacitinib	46.44 ± 14.12	49 ± 16.471	0.43*	0.487
Gender				
Female	6 (66.7)	36 (87.8)	2.45	0.117
Male	3 (33.3)	5 (12.2)		
Duration of disease (years)	10 ± 6.02	10.61 ± 7.1	5	0.518
BMI				
< 24.9	4 (44.4)	6(14.7)		
25-29.9	0 (0.0)	16 (39)	7.44	0.19
≥ 30	5 (66.6)	19 (46.4)		
Comorbidities				
No	2 (22.2)	15 (36.6)	0.67	0.41
Yes	7 (77.8)	26 (63.4)		
Serology				
Seronegative	3 (33.3)	4 (9.8)	3.4	0.065
Seropositive	6 (66.7)	37 (90.2)		
Presence of erosion				
No	7 (77.8)	28 (68.3)	0.31	0.574
Yes	2 (22.2)	13 (31.7)		
Extra-articular manifestation				
None	8 (88.9)	35 (85.4)	0.07	0.783
Yes	1 (11.1)	6 (14.6)		
Tofacitinib as monotherapy				
No	7 (77.8)	27 (65.9)	0.48	0.487
Yes	2 (22.2)	14 (34.1)		
Given after a biological agent				
No	1 (11.1)	12 (29.3)	1.26	0.261
Yes	8 (88.9)	29 (70.7)		
Number of previous csDMARDs	1.44 ± 0.72	1.51 ± 0.55	0.56	0.619
Number of previous bDMARDs	1.89 ± 1.36	1.22 ± 1.01	0.18	0.211

## Discussion

This retrospective real-world study analyzing patients with RA treated with tofacitinib from two centers in Western Saudi Arabia showed a good response to tofacitinib treatment even without concomitant use of MTX and in patients who had multiple bDMARD failure. Tofacitinib significantly reduced disease activity, with 82% achieving the treatment target at follow-up. In the clinical trials, the treatment target was achieved according to DAS28-ESR in up to 47.5% [13-15] while in real-world studies, it is up to 58% [16-17].

Tofacitinib was discontinued by 14% of the patients due to ineffectiveness, which is lower than the results of other clinical studies [15, 18]. In an open-label study, 20.4% of patients did not achieve ACR 20 after 24 months [19]. In real-life data, discontinuation due to ineffectiveness ranges from 14% to 68% [17]

Tofacitinib failure was not predicted by any baseline characteristics at the time of starting tofacitinib therapy. In our study, 26% of the patients were biologic-naïve. There was no significant difference in response whether patients were naïve or had been exposed to biological agents. Serologic status has no impact on treatment outcome. The likelihood of achieving the treatment target for tofacitinib was higher in biologic-naïve patients in many studies of bDMARDs [20-21]. In a retrospective Turkish single-center study, it was found that negative RF was an independent predictor of a good response to tofacitinib [22]. However, there is no conclusive evidence on the relationship between serologic status and tofacitinib response.

Adverse events leading to discontinuation of tofacitinib treatment were observed in 10% of the patients which was comparable to published data. The reported rate of discontinuation due to adverse events ranges from 4% to 26% in observational studies [17]. In our study, the most common infectious adverse events were herpes zoster (4%), pneumonia (2%), and complicated UTI (2%). In a three-year study post-marketing surveillance of tofacitinib in Japan, 3.7% developed herpes zoster [23]. The incidence rates of herpes zoster and other infections in a retrospective Turkish single-center study were found to be 3.9 and 1.4 per 100 patient years, respectively [22].

Few patients (6%) developed an increase in LDL cholesterol following the initiation of tofacitinib. It has been reported that the treatment with tofacitinib is associated with an elevation in total cholesterol, LDL, and HDL; however, it does not appear to be associated with an increased cardiovascular risk [23].

Neither hepatitis B virus (HBV) reactivation nor tuberculosis was reported. None of the enrolled patients had VTE, MACE, or malignancy; however, the follow-up period was not enough to make a conclusion. Several limitations of this study must be mentioned such as the small number of participants. The duration of the study is insufficient to capture serious adverse events such as MACE, malignancy, and VTE.

## Conclusions

Tofacitinib is a valuable and well-tolerated treatment option for RA patients. Tofacitinib induced high rates of low disease activity and remission in moderate to highly active RA as monotherapy or in combination with csDMARDs, which reinforces tofacitinib effectiveness in real clinical practice.
